# COVID-19 vaccine hesitancy in KwaZulu-Natal, South Africa: A survey of unvaccinated adults

**DOI:** 10.4102/hsag.v29i0.2468

**Published:** 2024-02-01

**Authors:** Tanuja N. Gengiah, Donavan C. Naidoo, Nomcebo Maduma, Saien Govender, Sherishka Dhindayal, Lara Lewis

**Affiliations:** 1 Center for the AIDS Programme of Research in South Africa (CAPRISA), Durban, South Africa; 2 Discipline of Pharmaceutical Sciences, School of Health Sciences, University of KwaZulu-Natal, Durban, South Africa; 3 Discipline of Pharmaceutical Sciences, School of Health Sciences, University of KwaZulu-Natal, Durban, South Africa

**Keywords:** COVID-19, vaccine, hesitancy, vaccination, survey, South Africa

## Abstract

**Background:**

Concerns and misconceptions surrounding coronavirus disease 2019 (COVID-19) vaccines may account for vaccine hesitancy and low uptake.

**Aim:**

To determine prevalence of COVID-19 vaccine hesitancy, vaccine-related misconceptions, and predictors of vaccine hesitancy among South Africans.

**Setting:**

Community setting in five districts in KwaZulu- Natal province.

**Methods:**

Between August 20, 2021, and September 27, 2021, we conducted a cross-sectional survey, interviewing 300 unvaccinated adults amid the national vaccination campaign. Predictors of hesitancy were identified through multivariable logistic regression analysis.

**Results:**

Participants had a median age of 29 years (IQR: 23–39), 86.7% were Black African, 63.2% were male, 53.3% resided in rural communities, and 59.3% (95% CI: 53.8% – 64.9%) were classified as vaccine hesitant. The primary reason for not vaccinating was a lack of trust in the vaccine (62.1%). Factors associated with reduced vaccine hesitancy included age (participants aged 35–49 years: OR: 0.28, 95% CI: 0.18–0.64, *p* = 0.003; participants over 50 years: OR: 0.18, 95% CI: 0.07–0.47, *p* = 0.0004), previous COVID-19 infection (OR: 0.31, 95% CI: 0.11–0.87, *p* = 0.03), and receiving vaccine information from healthcare workers (OR: 0.32, 95% CI: 0.10–1.0, *p* = 0.05). Unemployed (OR: 2.14, 95% CI: 1.1–4.2, *p* = 0.03) and self-employed individuals (OR: 2.98, 95% CI: 1.27–7.02, *p* = 0.01) were more likely to be vaccine hesitant.

**Conclusion:**

COVID-19 vaccine hesitancy rates are high in KwaZulu-Natal. Uptake could be enhanced by healthcare workers leading information campaigns with messages targeting younger individuals, the unemployed, and the self-employed.

**Contribution:**

This survey provides evidence to improve COVID-19 vaccination uptake in South Africa.

## Introduction

The coronavirus disease 2019 (COVID-19) pandemic was first classified as a public health emergency of international concern (PHEIC) by the World Health Organization (WHO) in January 2020 (WHO 2020). By the end of March 2023, COVID-19 was responsible for over 6.8 million deaths worldwide of which over 102 000 were reported in South Africa (WHO 2023). While various non-pharmaceutical interventions, such as the strict countrywide lockdowns, use of facemasks, social distancing, hand sanitisation, and isolation of COVID-19 patients have been used to combat the spread of severe acute respiratory syndrome coronavirus 2 (SARS-CoV-2) (Li et al. 2021; Perra 2021), it has been the COVID-19 vaccine, regardless of the type, that has most effectively reduced morbidity and mortality globally (Johnson & Stobbe 2021; Huang & Kuan 2022). The benefits of vaccination against COVID-19 include protection from severe disease, reduced mortality rates, and reduced need for hospitalisation while offering protection to others by reducing the onward transmission of COVID-19 (Polack et al. 2020; Sadoff et al. 2021).

In South Africa, the COVID-19 vaccine first became available in February 2021 with 1.2 million frontline healthcare workers receiving priority access to the Johnson and Johnson (Ad26.COV2.S) vaccine (Powell 2021). As of 20 March 2023, 25 months since the vaccine became available in South Africa, over 19 million South African adults (19 430 293) have been fully vaccinated against COVID-19 (South African National Department of Health 2023). However, despite the approval and availability of COVID-19 vaccines in South Africa, as well as the availability of existing data on the safety and efficacy of these vaccines (Polack et al. 2020; Sadoff et al. 2021), only 49.24% of the adult population has been fully vaccinated (South African National Department of Health 2023). As of 20 March 2023, KwaZulu-Natal (KZN) remains the province with the lowest vaccination rate of 35.64% in comparison with the other eight provinces. From the 49.24% of total vaccinated individuals of South Africa, most were aged 60 and over (66.74%) (South African National Department of Health 2023).

Vaccine hesitancy, defined as the delay in the acceptance or refusal of vaccination despite its availability (Jacobson, St. Sauver & Finney Rutten 2015), can be seen as a continuum ranging from complete acceptance to complete refusal of a specific vaccine or of vaccination in general (Larson et al. 2011). Numerous factors, such as complacency (low risk perception), a lack of confidence in the vaccine (by expressing concerns over the efficacy and safety), and convenience, can influence vaccine hesitancy (Bedford et al. 2018) and in turn determine the success or failure of any vaccination programme (Freeman et al. 2022).

To better support the COVID-19 vaccination programme in South Africa, it is crucial to identify factors associated with vaccine hesitancy, to debunk associated myths and misconceptions, and recognise predictors of hesitancy. This study aimed to assess the prevalence of vaccine hesitancy, identify predictors, and understand misconceptions surrounding the COVID-19 vaccine.

## Research methods and design

### Study design

The study design included a cross-sectional community-based survey assessing the prevalence of COVID-19 vaccine hesitancy and vaccination-related concerns in KZN, South Africa.

### Study setting

Data were collected from participants in five districts, viz.: eThekwini, Zululand, uMzinyathi, uMkhanyakude, and uGu located in the KZN province during the period 20 August–27 September 2021. At the time of this study, the South African National Department of Health was offering access to vaccination using a phased approach prioritising certain groups of the population (healthcare workers, essential workers, people older than 60 years, adults with comorbidities, and people in congregate settings) based on risk criteria for increased morbidity and mortality if infected with SARS-CoV-2 (South African National Department of Health 2021).

### Study population and sampling strategy

Unvaccinated adults (> 18 years of age) from both rural and urban areas, who provided written informed consent were eligible for study inclusion. Survey participants were approached at random and recruited through street recruitment at transport hubs, taxi ranks, and shopping centres. The adult population of South Africa was estimated to be 43 million. To achieve a precise estimate, a margin of error of approximately 5.5% was deemed acceptable for the survey. Assuming a vaccine hesitancy rate of approximately 30% (Cooper, Van Rooyen & Wiysonge 2021; Engelbrecht, Heunis & Kigozi 2022; Wiysonge et al. 2021), a sample size of 300 participants was necessary to achieve a 95% confidence interval with the desired interval width of 11%.

### Data collection

A structured questionnaire was used to collect information on socio-demographic characteristics, respondents’ perceptions about COVID-19, prevalence of vaccine hesitancy and misconceptions surrounding COVID-19 vaccination. The questionnaire was designed by researchers after reviewing previously used survey questionnaires (Bohler-Muller et al. 2021; Runciman et al. 2021). The questionnaire was then adapted to answer the current research objectives. While no statistical tests were applied to assess for internal consistency, the instrument was reviewed by a behavioural scientist and pilot tested among the researchers prior to finalisation.

A unique identifier was assigned to each questionnaire and all responses were anonymised. Final year pharmacy students from the Discipline of Pharmaceutical Sciences at the KZN conducted the face-to-face interviews of consenting study participants. To ensure ethical conduct and consistent data collection, student interviewers were mandated to obtain online ethics training certification from Training and Resources in Research Ethics Evaluation (TRREE). They underwent training to conduct interviews in a standardised manner, ensuring uniformity and adherence to ethical guidelines.

### Data analysis

Data were entered on paper surveys in real-time and then captured electronically in a REDCap® database by the interviewers. The data were exported and analysed using SAS version 9.4 (SAS Institute, Cary, North Carolina). Descriptive statistics were used to describe participant socio-demographic data. To assess vaccine hesitancy, participants were asked ‘Would you take the COVID-19 vaccines if it was available to you right now?’ to which they could answer ‘Yes’, ‘No’ or ‘Unsure’. Those who responded ‘No’ or ‘Unsure’ were classified as vaccine hesitant. A Fisher’s exact test (univariable) and a multivariable logistic regression model was performed to assess predictors for vaccine hesitancy. All variables that were significant in the univariable analysis as well as variables of interest that proved to be associated with vaccine hesitancy were included in the multivariable analysis. The resulting estimates are reported as odds ratio estimates, their corresponding 95% confidence intervals, and a corresponding *p*-value. A *p*-value less than 0.05 was considered statistically significant.

## Results

Participants had a median (interquartile range [IQR]) age of 29 (23–39) years and the majority were black African (86.7%) and male (62.7%). *IsiZulu* was the most common home language spoken by 233 (77.7%) participants and 160 (53.3%) resided in a rural area (Table 1).

**TABLE 1:** Socio-demographic characteristics of survey respondents (*N* = 300).

Variable	Median	IQR	*n*	%
**Age (years)**	29	23–39	-	-
Population group	-	-	-	-
Black African	-	-	260	86.7
Indian or Asian	-	-	31	10.3
White	-	-	5	1.7
Mixed race	-	-	4	1.3
**Gender**				
Male	-	-	188	62.7
Female	-	-	112	37.3
**Home language**				
IsiZulu	-	-	233	77.7
English	-	-	42	14.0
Xhosa	-	-	10	3.3
Sotho	-	-	7	2.3
Afrikaans	-	-	3	1.0
Swati	-	-	2	0.7
Tsonga	-	-	1	0.3
Venda	-	-	1	0.3
Pedi	-	-	1	0.3
**Education level attained**				
No formal schooling	-	-	16	5.3
Primary	-	-	17	5.7
Secondary	-	-	152	50.7
Tertiary	-	-	115	38.3
**Occupation**				
Unemployed	-	-	87	29.0
Employed	-	-	85	28.3
Student	-	-	70	23.3
Self-employed	-	-	41	13.7
Pensioner or retired	-	-	17	5.7
**Place of residence area**				
Rural	-	-	160	53.3
Urban formal	-	-	113	37.7
Urban informal dwelling	-	-	24	8.0
Traditional	-	-	3	1.0
**Dependents**				
Yes	-	-	162	54.0
No	-	-	138	46.0
Number of dependents median	-	1–5	3	-

IQR, interquartile range.

Of the 300 participants interviewed, 19 (6.3%) self-reported previous COVID-19 infection, 113 (37.7%) reported knowledge of a previous COVID-19 infection among family members or close friends, and 106 (35.3%) participants reported that they personally knew of people who had died because of COVID-19 disease.

**FIGURE 1:**
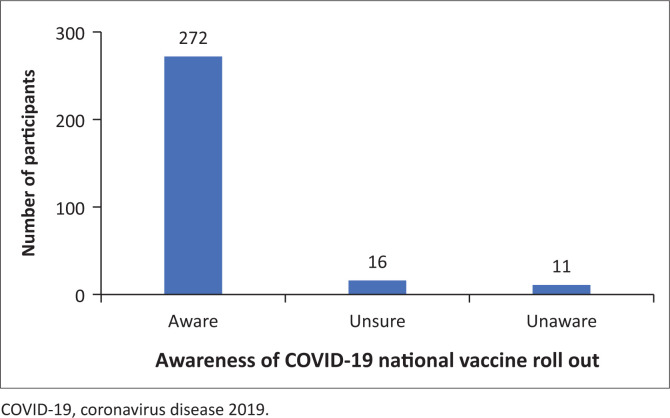
Awareness of coronavirus disease 2019 vaccine availability.

Most participants (90.7%) were aware that COVID-19 vaccines were available to the public in a phased approach (Figure 1).

The prevalence of vaccine hesitancy was estimated to be 59.3% (95% CI: 53.8%–64.9%) (*n* = 178); where 124 (41.3%) and 54 (18%) of participants, respectively, responded that they would either refuse the vaccine, or were unsure about vaccination, if the vaccine was immediately available to them. When asked about their main sources of COVID-19 information; social media (e.g., Facebook, Instagram, and Twitter) was the most frequently reported source by 134 (44.7%) of survey participants, followed by television broadcasts from the Presidency or Minister of Health reported by 120 (40%) respondents and 5% cited healthcare workers as a primary source of information.

Participants provided various reasons for their willingness to receive a COVID-19 vaccine, and all relevant reasons were documented (see Table 2). The primary motivation cited was self-protection, with 77% of participants indicating this as their main reason.

**TABLE 2:** Reasons participants were willing to receive the coronavirus disease 2019 vaccine if offered (*N* = 122).

Reasons for acceptance	*n*	%
To protect themselves against the virus	94	77.0
Fear of contracting or dying from COVID-19	53	43.4
Fear of loved ones contracting or dying from COVID-19	48	39.3
Protect others by preventing viral transmission	39	32.0
Wants to get back to normal life	34	27.9
Wants to stop wearing a mask all the time	27	22.1
Encouraged by others who had already taken the vaccine	12	9.8
**Other reasons for willingness to receive a COVID-19 vaccine:**	12	9.8
Fear of being restricted access to shops, liquor stores, stadiums, clinics, social interaction, or travelling in general without proof of vaccination	7	-
Compelled to take the vaccine because of work regulations	4	-
Fear of being denied a job opportunity because of being unvaccinated	1	-
Trusts the COVID-19 vaccine and vaccines in general	9	7.4
Fear of death because of existing co morbidities (e.g. high blood pressure, obesity, diabetes)	4	3.3

COVID-19, coronavirus disease 2019.

Participants expressed several vaccine-related concerns that led to their refusal or their uncertainty to receive a COVID-19 vaccine (Table 3 and Table 4). For participants who were hesitant about receiving the vaccine when offered, the main reason cited was a lack of trust in the vaccine’s effectiveness (62.1%). Additionally, 51.6% expressed a lack of trust in the government that procured the vaccine, and 38% reported fear of adverse reactions related to the vaccine.

**TABLE 3:** Reasons participants *would not* receive the vaccine if offered (*N* = 124).

Reasons for refusal	*n*	%
Does not trust the COVID-19 vaccine	77	62.1
Does not trust the government who procured the vaccine	64	51.6
Fear of vaccine related adverse reactions (e.g., blood clots, fever, body pain)	38	30.6
Fear of contracting COVID-19 from the vaccine	26	21.0
Concerns about the short duration of time in which the vaccines were produced	24	19.4
Can protect themselves without a vaccine	24	19.4
Does not trust vaccines in general	20	16.1
Heard rumours that the vaccine contains a microchip to track the population	17	13.7
Personal reasons (not elicited)	17	13.7
Religious beliefs preclude vaccination	13	10.5
**Other reasons for not being willing to receive a COVID-19 vaccine:**	12	9.8
Pre-existing allergies to several medications	1	-
Felt that they did not need the vaccine because they are not immunocompromised or sick	2	-
Felt that COVID-19 vaccines do not work	2	-
Feared that the vaccine may be implicated in the post vaccination deaths of family members or others known to them	4	-
Felt that exercise supplements provided immunity against COVID-19	1	-
Felt that traditional medicine would protect them	1	-
Unanswered questions about the importance of the COVID-19 vaccine	1	-
Fear of needles	11	8.9
Fear of vaccines altering their DNA	6	4.8

COVID-19, coronavirus disease 2019.

**TABLE 4:** Reasons participants were unsure about receiving the coronavirus disease 2019 vaccine (*N* = 54).

Reasons for uncertainty	*n*	%
Fear of vaccine-related adverse reactions (e.g., blood clots, fever, body pain)	29	53.7
Concerns about the short duration of time that the vaccines were produced	16	29.6
Sceptical about vaccines in general	13	24.1
**Other reasons participants are unsure about receiving the COVID-19 vaccination:**	12	9.8
Vaccine mistrust because it is manufactured overseas	1	-
Felt that vaccine production was focused more on competition than reproducibility	1	-
Fear of being unable to conceive after taking the vaccine	1	-
Fear that vaccines were made to kill people	1	-
Underlying conditions have weakened immunity	1	-
Fear of dying after taking the vaccine and leaving children behind	1	-
Religious beliefs preclude vaccination	8	14.8
Want protection against the virus, but there is fear of contracting COVID-19 from the vaccine	8	14.8
Heard rumours that the vaccine contains a microchip to track the population	6	11.1
Fear about the vaccine cost	4	7.4
Fear of vaccines altering their DNA	3	5.6
Fear of needles	1	1.9

COVID-19, coronavirus disease 2019.

Individuals who were uncertain about taking the vaccine expressed primary concerns about potential adverse effects and were worried about the relatively short production time of the vaccines (29.6%) (Table 4).

To assess predictors for vaccine hesitancy a stepwise regression model was built (Table 5). Participants who were unsure about being vaccinated or refused vaccination were combined and assigned in the model as ‘vaccine hesitant’. This was because age is strongly associated with occupation; pensioners and the unemployed were grouped together.

**TABLE 5:** Univariate regression model using the Fisher’s exact test.

Variable	Would get vaccinated (*n* = 122, 40.7%)				Unsure or refused vaccination* (*n* = 178, 59.3%)				*p*
	Median	IQR	*n*	%	Median	IQR	*n*	%	
**Age**	34	24–66	-	-	27	23–34	-	-	< 0.001*
**Age (in 10-year intervals)**									
18–19	-	-	7	33.3	-	-	14	66.7	-
20–29	-	-	39	29.5	-	-	93	70.5	-
30–39	-	-	31	42.5	-	-	42	57.5	-
40–49	-	-	20	58.8	-	-	14	41.2	-
50–59	-	-	17	70.8	-	-	7	29.2	-
60+	-	-	8	50.0	-	-	8	50.0	-
**Gender**									0.466
Female	-	-	49	43.8	-	-	63	56.3	-
Male	-	-	73	38.8	-	-	115	61.2	-
**Education**									0.849
Primary or less	-	-	15	45.5	-	-	18	54.5	-
Secondary	-	-	61	40.1	-	-	91	59.9	-
Tertiary	-	-	46	40.0	-	-	69	60.0	-
**Occupation**									0.119
Unemployed	-	-	33	37.9	-	-	54	62.1	-
Student	-	-	23	32.9	-	-	47	67.1	-
Self-employed	-	-	14	34.1	-	-	27	65.9	-
Employed	-	-	44	51.8	-	-	41	48.2	-
Pensioner or retired	-	-	8	47.1	-	-	9	52.9	-
**Place of residence**									0.099
Rural	-	-	59	36.2	-	-	104	63.8	-
Urban	-	-	63	46.0	-	-	74	54.0	-
**Has dependents**									0.060
No	-	-	48	34.8	-	-	90	65.2	-
Yes	-	-	74	45.7	-	-	88	54.3	-
**Had previous COVID- 19 infection**									0.052*
No	-	-	110	39.1	-	-	171	60.9	-
Yes	-	-	12	63.2	-	-	7	36.8	-
**Knows of friend or family with COVID-19 infection**									0.904
No	-	-	77	41.2	-	-	110	58.8	-
Yes	-	-	45	39.8	-	-	68	60.2	-
**Reported known COVID-19 death**									0.176
No	-	-	73	37.6	-	-	121	-	-
Yes	-	-	49	46.2	-	-	57	53.8	-
**Source of COVID-19 health information**									
Newspaper media	-	-	16	34.8	-	-	30	65.2	0.418
Social media	-	-	54	40.3	-	-	80	59.7	1.000
Internet	-	-	19	35.2	-	-	35	64.8	0.445
Lecturers or teachers	-	-	1	20.0	-	-	4	80.0	0.652
Family and friends	-	-	16	37.2	-	-	27	62.8	0.738
President or health minister in live TV broadcasts	-	-	56	46.7	-	-	64	53.3	0.094
Pharmacist or doctor or nurse	-	-	9	60.0	-	-	6	40.0	0.176
Other source (radio and workplace)	-	-	15	48.4	-	-	16	51.6	0.440

* , Statistically significant; IQR, interquartile range.

All variables that were significant in the univariate analysis were included in the multivariate analysis (Table 6). In addition, variables of interest that have been shown to be associated with vaccine hesitancy were also included in the model.

**TABLE 6:** Multivariable logistic regression model measuring association between factors and vaccine hesitancy (*N* = 300).

Variable (*Reference group*)	Effect	Adjusted odds ratio estimates	95% confidence intervals		*p*
Age (years) (*< 25 years*)	25–34	0.921	0.413	2.055	0.8414
	35–49	0.275	0.117	0.644	0.0030*
	50+	0.177	0.068	0.465	0.0004*
Gender (*Female*)	Male	1.252	0.745	2.105	0.3964
Place of residence (*Urban*)	Rural	1.443	0.866	2.404	0.1590
Occupation (*Employed*)	Self-employed	2.983	1.267	7.021	0.0123*
	Student	1.244	0.506	3.06	0.6341
	Unemployed or retired	2.141	1.094	4.191	0.0263*
History of COVID-19 infection (*No*)	Yes	0.308	0.109	0.868	0.0260*
Source of COVID-19 information (*Other sources*)	Received COVID-19 information from pharmacist or doctor or nurse	0.320	0.101	1.011	0.0522*

COVID-19, coronavirus disease.

* , Statistically significant.

## Discussion

This cross-sectional study among adults in KZN demonstrated that 59.3% of participants were unsure or would refuse COVID-19 vaccination. Age, occupation, history of COVID-19 infection, and the source of COVID-19 information had a significant association with vaccine hesitancy. The main reasons for refusal to be vaccinated were a lack of trust in the COVID-19 vaccine itself and in the government who procured the vaccine, while the main reasons for uncertainty included the fear of vaccine-related adverse reactions and concerns about the short duration of time that the vaccines were produced. Interestingly, the main sources of COVID-19 information for this sample of participants were social media (e.g., Facebook, Instagram, and Twitter) and information given by the president or health minister on the news in contrast to vaccine acceptors who received vaccine-related information from healthcare workers such as doctors, nurses, or pharmacists.

Studies conducted in South Africa have previously shown COVID-19 vaccine hesitancy among the general population (Cooper et al. 2021). However, our study had a higher prevalence of vaccine hesitancy compared to previous reports ranging from 24% in September 2020 to 30% in February 2021 and (Burger et al. 2022; Cooper et al. 2021; Wiysonge et al. 2021). Previous surveys in South Africa were conducted online among a selected population with access to smartphone technology resulting in significantly larger sample sizes (Engelbrecht et al. 2022; Runciman et al. 2021). Our survey stood out because of its grassroots approach, involving in-person interviews conducted during the day at locations such as transport hubs, taxi ranks, and shopping centres. However, the smaller sample size in our study could have influenced the primary findings. It is worth observing that approximately a third of the participants were unemployed (29%) and the median age of those classified as vaccine hesitant was 27 years. In our study, age was significantly associated with vaccine hesitancy because younger people were less likely to accept the vaccines. This finding is in line with research from South Africa, India, and Nigeria where vaccine hesitancy was also higher among younger people (Engelbrecht et al. 2022; Solis Arce et al. 2021). Using 10-year intervals the authors found that the highest proportion of vaccine hesitant individuals was in the age group 20–29 years (70.5%). Consistent with the University of Johannesburg Human Sciences Research Council (UJ-HSRC) study (Runciman et al. 2021), the percentage of those who would definitely or probably get the vaccine was highest among those aged over 55 years (74%) and lower in younger age groups.

There are conflicting reports on the association of gender and COVID-19 vaccine hesitancy. This study found no gender-based differences in vaccine hesitancy. While there might have been fewer women than men in this study, making it challenging to demonstrate significant differences, other studies have indicated that men are more likely to exhibit vaccine hesitancy (Babalola et al. 2020, Runciman et al. 2021), while data from the Africa Centres for Disease Control and Prevention (CDC) (2021) showed that willingness to accept the COVID-19 vaccine is similar across genders (73% of men vs. 78% of women). In this study, vaccine hesitancy increased with the level of education, in line with other findings (Engelbrecht et al. 2022; Runciman et al. 2021); however, these data did not reach statistical significance. Furthermore, participants who were employed reported to be more likely to get vaccinated than those who are unemployed or self-employed. This could be attributed to the impact of restricted movement and lockdowns on the informal business sector, which eroded the trust in government and influenced vaccine uptake (Paul, Fancourt & Razai 2022)

The main reasons for vaccine hesitancy in the South African setting, evident in this study and supported by others (Cooper et al. 2021; Runciman et al. 2021), were related to concerns about safety and effectiveness of the vaccine and the potential for side effects. Other concerns included short duration of time that vaccines were produced, and a lack of trust in authorities promoting the vaccine, also reported by others (Bogart et al. 2021; Katoto et al. 2022). It is important to recognise the fact that when there is low trust in the government and incongruence between social media (high possibility for misinformation) and local media reports, then vaccine refusal and hesitancy would be high as reported in Malaysia (Chan et al. 2022). Trusted sources of health information, particularly in pandemics, need to be established on platforms that are easily and commonly accessed by communities with healthcare workers playing a more prominent role.

In our study, the authors showed that individuals with a history of previous COVID-19 infection were more likely to accept the vaccine. This observation has been echoed in other studies (Engelbrecht et al. 2022; Runciman et al. 2021) suggesting that having a history of COVID-19 infection or observing the affected is associated with higher vaccine acceptance. This could be attributed to the motivation to avoid reinfection or to reduce the severity of illness based on firsthand experience. Information sources had an impact on COVID-19 vaccine hesitancy in this study and participants who received COVID-19 health-related information from healthcare workers were less likely to be vaccine hesitant. It has been shown previously that prevalence of COVID-19 vaccine hesitancy among the participants was lowest among newspaper readers (42%) and highest among TV (72%) and social media users (73%) and obtaining information from healthcare workers had a positive influence on intent towards vaccination in other sub-Saharan African settings (Osuagwu et al. 2023).

This study has several limitations. The authors could not distinguish between vaccine hesitancy and vaccine denial. While efforts were made to guard against socially acceptable responses, this was not completely avoided. With street recruitment, refusal to participate was common and several potential participants declined the survey once they realised that they needed to sign an informed consent form. Many young women were not comfortable to stop and take the survey and this resulted in more men being sampled.

Despite these limitations, this community-based survey provided useful information on the prevalence of vaccine hesitancy and documented the misconceptions about the COVID-19 vaccine use in KZN, South Africa. This study showed that social media plays an important role in influencing the way people perceive the COVID-19 vaccines. Reliable, professional sources of health information should be introduced on social media platforms to inform the public about accurate COVID-19 vaccine information and to provide support.

Community concerns can be allayed, and vaccine uptake encouraged by developing platforms where other community members can share their first-hand experiences with COVID-19 vaccination.

The public should be educated about expected symptoms following injection administrations, for example fever, body pain and that they are mostly mild, manageable, and short-lived to address concerns about potential side effects from vaccines. As people are more likely to trust advice received from their healthcare providers, there is a need for healthcare providers to campaign, and offer trustworthy advice to the public. The latter could counteract scepticism and vaccine hesitancy if an evidence-based approach of motivational interviewing to improve vaccine uptake behaviours is used.

## Conclusion

A high rate of vaccine hesitancy was observed among adults in KwaZulu-Natal, South Africa. Among those most likely to exhibit hesitancy are younger individuals, self-employed and unemployed individuals, those without previous COVID-19 infection, and those who obtain vaccine information from non-healthcare sources. To improve vaccine uptake, health authorities should prioritise these specific groups when implementing information campaigns to educate the public about COVID-19 vaccination. Utilising social media platforms and digital channels would be useful for reaching and engaging younger individuals.
